# Insights into the Biocontrol Function of a Burkholderia gladioli Strain against Botrytis cinerea

**DOI:** 10.1128/spectrum.04805-22

**Published:** 2023-03-02

**Authors:** Dan Wang, Wan-Zhen Luo, Dan-Dan Zhang, Ran Li, Zhi-Qiang Kong, Jian Song, Xiao-Feng Dai, Noam Alkan, Jie-Yin Chen

**Affiliations:** a State Key Laboratory for Biology of Plant Diseases and Insect Pests, Institute of Plant Protection, Chinese Academy of Agricultural Sciences, Beijing, China; b Western Agricultural Research Center, Chinese Academy of Agricultural Sciences, Changji, China; c College of Agronomy, Xinjiang Agricultural University, Urumqi, China; d Department of Postharvest Science, Agricultural Research Organization, Volcani Institute, Rishon LeZion, Israel; Instituto de Ecología A.C.

**Keywords:** *Burkholderia gladioli*, *Botrytis cinerea*, biocontrol, plant growth promotion, plant immunity

## Abstract

Pathogenic fungi are the main cause of yield loss and postharvest loss of crops. In recent years, some antifungal microorganisms have been exploited and applied to prevent and control pathogenic fungi. In this study, an antagonistic bacteria KRS027 isolated from the soil rhizosphere of a healthy cotton plant from an infected field was identified as Burkholderia gladioli by morphological identification, multilocus sequence analysis, and typing (MLSA-MLST) and physiobiochemical examinations. KRS027 showed broad spectrum antifungal activity against various phytopathogenic fungi by secreting soluble and volatile compounds. KRS027 also has the characteristics of plant growth promotion (PGP) including nitrogen fixation, phosphate, and potassium solubilization, production of siderophores, and various enzymes. KRS027 is not only proven safe by inoculation of tobacco leaves and hemolysis test but also could effectively protect tobacco and table grapes against gray mold disease caused by Botrytis cinerea. Furthermore, KRS027 can trigger plant immunity by inducing systemic resistance (ISR) activated by salicylic acid- (SA), jasmonic acid- (JA), and ethylene (ET)-dependent signaling pathways. The extracellular metabolites and volatile organic compounds (VOCs) of KRS027 affected the colony extension and hyphal development by downregulation of melanin biosynthesis and upregulation of vesicle transport, G protein subunit 1, mitochondrial oxidative phosphorylation, disturbance of autophagy process, and degrading the cell wall of B. cinerea. These results demonstrated that B. gladioli KRS027 would likely become a promising biocontrol and biofertilizer agent against fungal diseases, including B. cinerea, and would promote plant growth.

**IMPORTANCE** Searching the economical, eco-friendly and efficient biological control measures is the key to protecting crops from pathogenic fungi. The species of Burkholderia genus are widespread in the natural environment, of which nonpathogenic members have been reported to have great potential for biological control agents and biofertilizers for agricultural application. Burkholderia gladioli strains, however, need more study and application in the control of pathogenic fungi, plant growth promotion, and induced systemic resistance (ISR). In this study, we found that a B. gladioli strain KRS027 has broad spectrum antifungal activity, especially in suppressing the incidence of gray mold disease caused by Botrytis cinerea, and can stimulate plant immunity response via ISR activated by salicylic acid- (SA), jasmonic acid- (JA), and ethylene (ET)-dependent signaling pathways. These results indicate that B. gladioli KRS027 may be a promising biocontrol and biofertilizer microorganism resource in agricultural applications.

## INTRODUCTION

Plant root and leaf diseases are significant threats to various crops worldwide, triggering severe economic and yield losses and threatening food security and human health. It is well known that pathogenic fungi are the dominant causal agents of plant diseases (1, 2). Gray mold disease, caused by Botrytis cinerea, is one of the most serious fungal diseases affecting flowers, fruits, leaves, and stems; it is difficult to control because the pathogen can survive in soil and plant residue for a long time (3, 4). B. cinerea is one of the most devastating postharvest diseases that cause gray mold during transportation and storage of tomato (Solanum lycopersicum), grapevine (Vitis vinifera L.), and over 200 other plant species and is responsible for significant direct losses that total 20% to 30% and even reach 50% in severe cases (Chinese research data) (5, 6). It is necessary to efficiently control the occurrence and spread of gray mold disease. Chemical-based pesticides are considered a simple and efficient control measure and are extensively applied in agriculture. However, long-term use of chemical pesticides can easily cause unsatisfactory pesticide residue levels, which would pollute the environment, threaten human and animal health, and increase drug resistance of pathogenic microorganisms (7, 8). Thus, the use of traditional chemical fungicides falls outside the concept of sustainable agriculture. In recent years, biological control has been widely studied and applied because it is economical, harmless, and eco-friendly (9).

Rhizosphere biocontrol microorganisms, also named antagonistic microorganisms, could have a significant role in the inhibition of plant-pathogenic microorganisms, play a part in plant growth promotion (PGP), and enhance plant resistance to biotic and abiotic stresses (10–13). An increasing number of antagonistic microorganisms have been discovered, studied, and applied in the control of fungal diseases, including various species of actinomycetes, Bacillus, Pseudomonas, Burkholderia, Klebsiella, Paenibacillus, etc. (14). For instance, Streptomyces philanthi RL-1-178 and RM-1-138 and Streptomyces mycarofaciens SS-2-243 showed a strong inhibition against sclerotium root and Ralstonia wilt of chili pepper and gray mold disease caused by B. cinerea (15). Bacillus species are known antagonistic microorganisms, including Bacillus subtilis, Bacillus amyloliquefaciens, Bacillus velezensis, Bacillus licheniformis, Bacillus pumilus, Bacillus tequilensis, etc., and have broad spectrum inhibitory activity against pathogenic fungi such as B. cinerea, Phytophthora spp., Colletotrichum spp., Fusarium spp., Verticillium spp., and Magnaporthe oryzae (16). Similarly, Burkholderia strains BE17 and BE24 isolated from the maize rhizosphere can inhibit the gray mold disease of grapevine (4, 17).

Not only antagonistic microorganisms but also their metabolic substances as extracellular secondary metabolites and volatile organic compounds (VOCs) are used as the source of biocontrol agents (18). A novel antimycin analogue isolated from Streptomyces sp. was active against Penicillium spp. and B. cinerea (19); 2,4-diacetylphloroglucinol and lipopeptides extracted from Pseudomonas bijieensis were active against bacterial canker and gray mold disease of kiwifruit (20). Various VOCs such as acetoin, acetic acid, 2,3-butanediol, isopentanol, dimethyl disulfide, and isopentyl isobutanoate released from extremophilic bacteria have effective biocontrol effects against postharvest fungal phytopathogens (21). Antagonistic microorganisms can also produce and secret indole acetic acid (IAA) and siderophores, induce phosphate/potassium solubilization and nitrogen fixation, and induce biofilm formation, which could promote plant growth (22). In addition, increasing evidence has indicated that beneficial rhizobacteria can induce plant systemic resistance (ISR) against biotic stresses, involving the activation of signaling networks as salicylic acid- (SA), jasmonic acid- (JA), and ethylene (ET)-dependent signaling pathways (13).

Burkholderia genus is a plant-beneficial bacteria that has become increasingly important due to its ability to inhibit pathogenic microorganisms by itself and by its derived antagonistic metabolites, including abundant extracellular secondary metabolites (bioactive compounds, siderophores, abundant enzymes as proteases, chitinases, amylase, cellulases, etc.) and VOCs, of which some species have been confirmed to promote plant growth and enhance plant resistance against biotic and abiotic stresses (23, 24). Several Burkholderia strains have been reported as potential biocontrol agents due to extremely antagonistic capacity and plant growth promotion. Members such as Burkholderia cepacia (25), Burkholderia seminalis (26), Burkholderia anthina (27), Burkholderia contaminans (28), Burkholderia pyrrocinia (29), and Burkholderia gladioli (30, 31) have been identified to inhibit phytopathogenic fungi. Moreover, Burkholderia sp. SSG was affirmed as a potential biofertilizer to promote boxwood growth (32). Burkholderia vietnamiensis displayed an ability to fix nitrogen and generate siderophore and indole acetic acid (IAA) (33). In addition, an increasing number of whole-genome sequences of Burkholderia strains were published. Comparative genomics analysis revealed the presence of gene clusters involved in bioactive secondary metabolite synthesis pathways, such as polyketide synthase (PKS) and nonribosomal peptide synthetase (NRPS), and plant growth promotion (PGP)-related genes also were found, which provided a certain basis for biocontrol mechanisms of antagonistic strains at the gene level (17, 30).

In this study, an antagonistic strain KR027 was isolated from the rhizosphere soil of a healthy plant that existed in a diseased cotton field from Xinjiang, China. To better understand this strain, a series of experiments were performed: taxonomic characterization was identified by morphological identification, MLST phylogenetic analysis, and physiobiochemical characteristics. Then, broad spectrum antifungal activity was evaluated against a variety of pathogenic fungi. In-depth antifungal analysis of the antagonistic strain KR027 was done against B. cinerea both *in vitro* and *in vivo*; evaluation for the innocuous and plant growth promotion and characterization of the inhibitory mode of action of KRS027 on B. cinerea growth and development were studied. The results of this study would provide the basis for potential biocontrol agents and biofertilizers in agriculture.

## RESULTS

### Morphological identification and phylogenetic analysis of B. gladioli strain KRS027.

**FIG 1:**
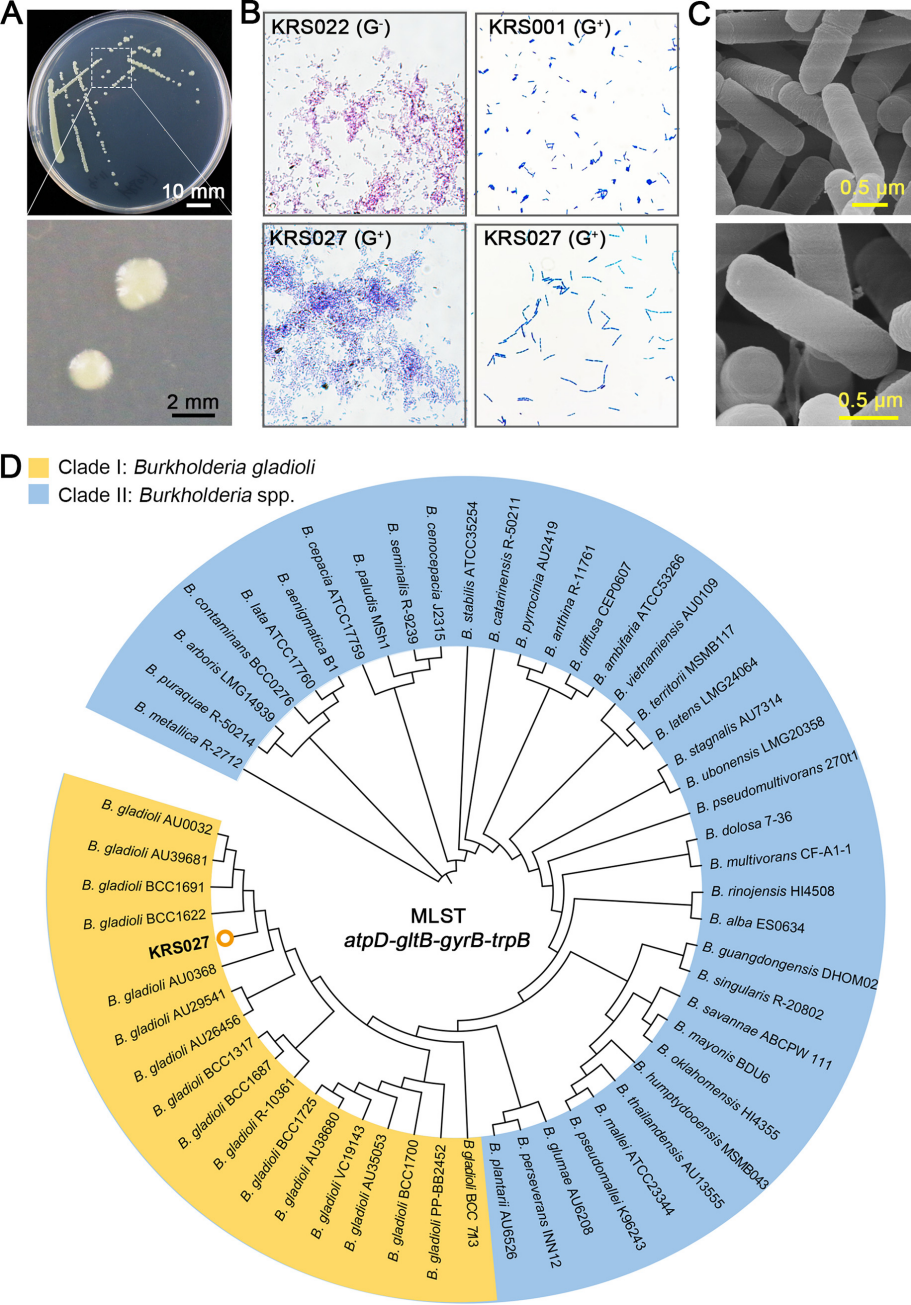
Morphological identification and phylogenetic analysis of strain KRS027. (A) The growth phenotype of strain KRS027 on Luria-Bertani (LB) medium. (B) Gram stain of Pseudomonas alcaligenes strain KRS022 and Bacillus velezensis KRS001 served as negative and positive controls, respectively. (C) Scanning electron microscopy (SEM) observation of strain KRS027. (D) Phylogenetic analysis for four housekeeping genes (*atpD*, *gltB*, *gyrB*, and *trpB*) tandem. The orange block represents Burkholderia gladioli, and the blue block represents other Burkholderia species. MLST, multilocus sequence typing.

B. gladioli strain KRS027 was isolated from the rhizosphere soil of a healthy plant found in a pathogen-infested cotton field from Xinjiang, China, and was found to have a high antifungal ability to inhibit fungal growth during a preliminary screening of antifungal antagonist microorganisms (our unpublished data). The single colony of KRS027 is oyster white, smooth-faced, nontransparent, and spherical after incubation for 24 h on Luria-Bertani (LB) agar plate (Fig. 1A). Isolate KRS027 was identified as a Gram-positive and coryneform bacterium by Gram staining (Fig. 1B). Scanning electron microscope (SEM) observation indicated that the KRS027 is a rod-shaped bacterium without flagella, and the ranges of length and width are 1.5 to 2.5 μm and 0.5 to 0.8 μm, respectively (Fig. 1C). A phylogenetic analysis was performed to identify strain KRS027. Although the 16S rRNA gene sequence of KRS027 had high similarity with B. gladioli based on the BLASTn from NCBI (data not shown), the multilocus sequence analysis and typing (MLSA-MLST) scheme for Burkholderia species has provided crucial insights for the population diversity (34, 35). A more refined sequence analysis involving MLSA-MLST (tandem loci as *atpD*, *gltB*, *gyrB*, and *trpB*) verified that the strain KRS027 belongs to the clade I group of B. gladioli (Fig. 1D).

### *In vitro* broad spectrum antifungal activity of isolate KRS027.

**FIG 2:**
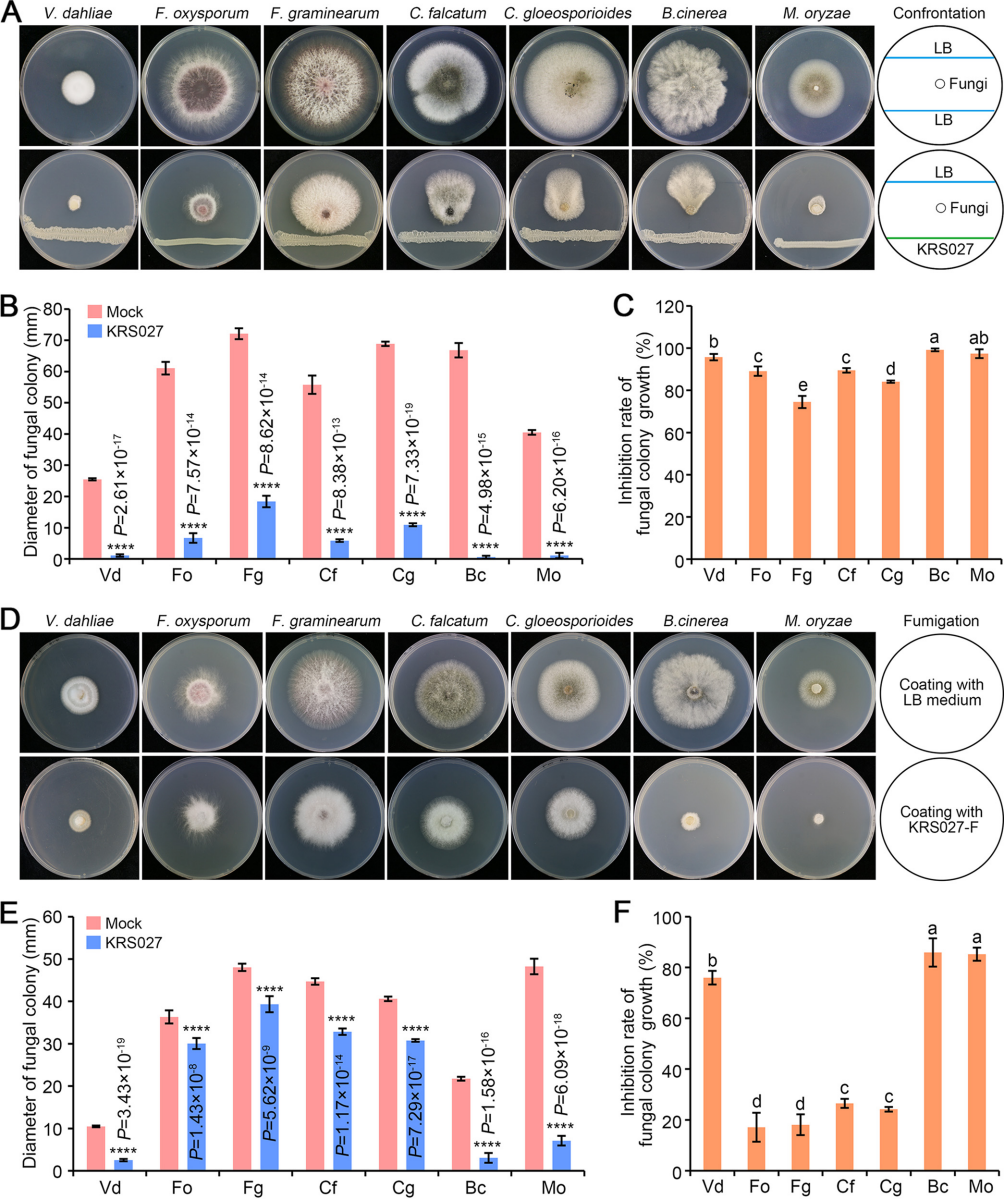
The broad spectrum inhibition activity of strain KRS027 against phytopathogenic fungi. (A) Antifungal activity of strain KRS027 against seven phytopathogenic fungi using confrontation culture assay. (B, C) The diameters of the fungal colony and the inhibition rate of fungal colony growth according to confrontation culture. (D) Antifungal activity of volatile organic compounds (VOCs) produced by KRS027 using the covering fumigation. (E, F) The diameters of fungal colony and inhibition rate of fungal colony growth by VOCs of KRS027. Error bars represent standard errors. ****, significant differences at *P* < 0.0001 according to unpaired Student’s *t* test. The letters (a to d) above columns represent the significant differences at *P* < 0.01 according to one-way analysis of variance (ANOVA) and least significant difference (LSD). Bc, Botrytis cinerea; Cf, Colletotrichum falcatum; Cg, Colletotrichum gloeosporioides; Fg, Fusarium graminearum; Fo, Fusarium oxysporum; Mo, Magnaporthe oryzae; Vd, Verticillium dahliae. KRS027-F, KRS027 fermentation broth.

It is well known that various plant-pathogenic fungi are responsible for the serious diseases of crops, including soilborne and airborne diseases. Seven of the more common and difficult-to-control filamentous pathogenic fungi were used to evaluate the broad spectrum antifungal activity of KRS027. The dual confrontation culture assays indicated that KRS027 had an inhibitory effect on these seven pathogenic fungi at different degrees (Fig. 2A). Most noteworthy is that colony growth of B. cinerea, Verticillium dahliae, and Magnaporthe oryzae were almost suppressed completely by isolate KRS027, which led to a suppression rate of more than 95%. The colony growth of B. cinerea reached 99.17%. In addition, the inhibition rates against Fusarium oxysporum, Fusarium graminearum, Colletotrichum gloeosporioides, and Colletotrichum falcatum were 80.66%, 58.58%, 73.43%, and 77.69%, respectively (Fig. 2B and C). The above results suggested that isolate KRS027 could secrete antifungal metabolites to exert suppression function.

The VOCs produced by microorganisms can affect pathogenic fungi colony growth, mycelial development, and conidia germination. Mycelial growth of all tested pathogenic fungi was strongly suppressed (B. cinerea, V. dahliae, and M. oryzae) or grew slower (F. oxysporum, F. graminearum, C. gloeosporioides, and C. falcatum) after fumigation with VOCs of KRS027 (Fig. 2D and E). The colony growth diameters of B. cinerea, V. dahliae, and M. oryzae were decreased by 85.90%, 75.99%, and 85.23%, respectively (Fig. 2F). Taken together, the inhibition function of VOCs produced by KRS027 significantly inhibited filamentous pathogenic fungi, especially in B. cinerea.

### *In vitro* PGP evaluation of KRS027.

**FIG 3:**
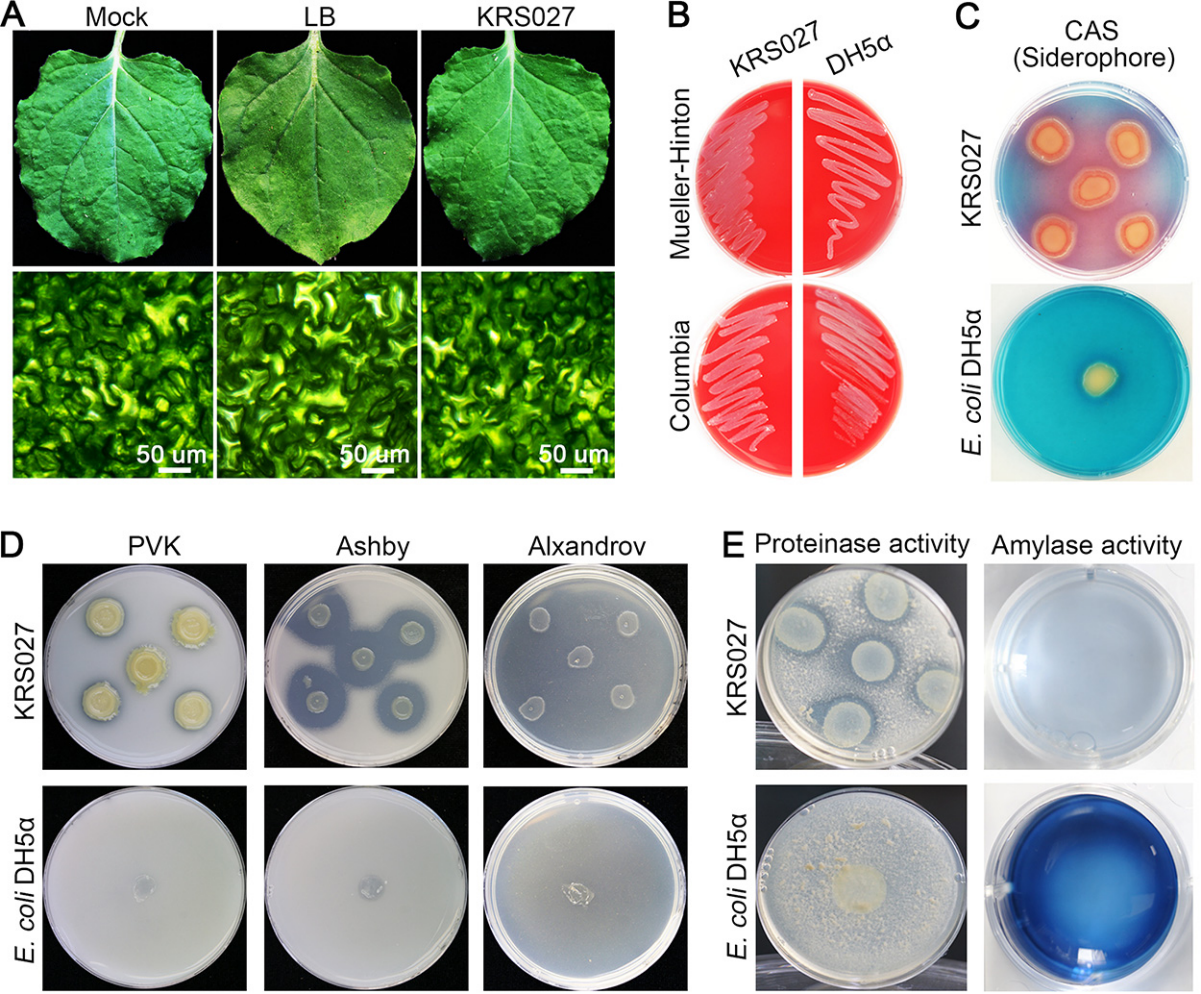
Potential effect of strain KRS027 on plants. (A) Safety evaluation of strain KRS027 on tobacco leaves. (B) Hemolysis test using Mueller-Hinton (MH) plates and Columbia Blood plates, and the E. coli DH5α was severed as control. (C) Siderophore production of KRS027 was determined using a blue agar medium containing Chrome Azurol S (CAS), and the yellow and red color changes represent siderophore production capacity of strain. (D) Nitrogen fixation and phosphate and potassium solubilization ability were determined by Ashby, Pikovskaya (PVK), and Alxandrov medium, respectively. (E) Proteinase and amylase activity assays. E. coli DH5α was severed as the negative control for all of the *in vitro* plant growth promotion (PGP) characteristic assessment assays.

The safety of isolates is an essential precondition for the potential biocontrol microbial application. The pathogenicity and hemolysis tests were performed to evaluate the safety of isolate KRS027. Nicotiana benthamiana leaves inoculated with isolate KRS027 did not show any disease symptom or necrosis; the mesophyll cells exhibited the same phenotype as the control by stereomicroscope observation (Fig. 3A). Taking human body safety into consideration, the hemolytic test was performed on Columbia and Mueller-Hinton blood plates. The results showed that the hemolytic ring was not observed around the colony of KRS027, which suggested that there is no α-hemolysin and that KRS027 does not break down red blood (Fig. 3B). Together, the above results suggested that KRS027 isolate is safe and innocuous.

*In vitro* assays involved in evaluating PGP were performed, which include siderophore production, phosphate/potassium solubilization, and nitrogen fixation. In this study, isolate KRS027 showed a promising plant growth-promoting effect. KRS027 could generate the red and yellow halo around the colony on blue Chrome Azurol S (CAS) plates manifesting its ability to produce siderophore (Fig. 3C). Macronutrients nitrogen (N), phosphorus (P), and potassium (K) are the three most essential nutrients for plant growth and development, whereas only minor portions of them are available for the plant. Taking the PGP features into account, strain KRS027 showed strong nitrogen-fixing activity and the ability to solubilize tricalcium phosphate and potash feldspar (Fig. 3D). In addition, other physiobiochemical characteristics suggested that the reactions for sulfur-containing amino acid utilization and nitrate reduction were positive. On the contrary, citrate utilization, gluconate utilization, methyl red assay, and indole production were negative (Table S2).

Destroying the phytopathogen’s cell wall is an important step for biocontrol bacteria to exert antifungal function. A variety of enzymes activities were detected, including gelatinase, proteinase, amylase, catalase, urease, and phenylalanine deaminase, which may help the isolate KRS027 to catalyze and hydrolyze the cell wall of pathogenic fungi (Fig. 3E; Table S2). Given the above observations, isolate KRS027 would likely become a very suitable candidate for further research and development as a potential biocontrol and biofertilizer strain.

### KRS027 protects the plant against gray mold disease caused by B. cinerea.

**FIG 4:**
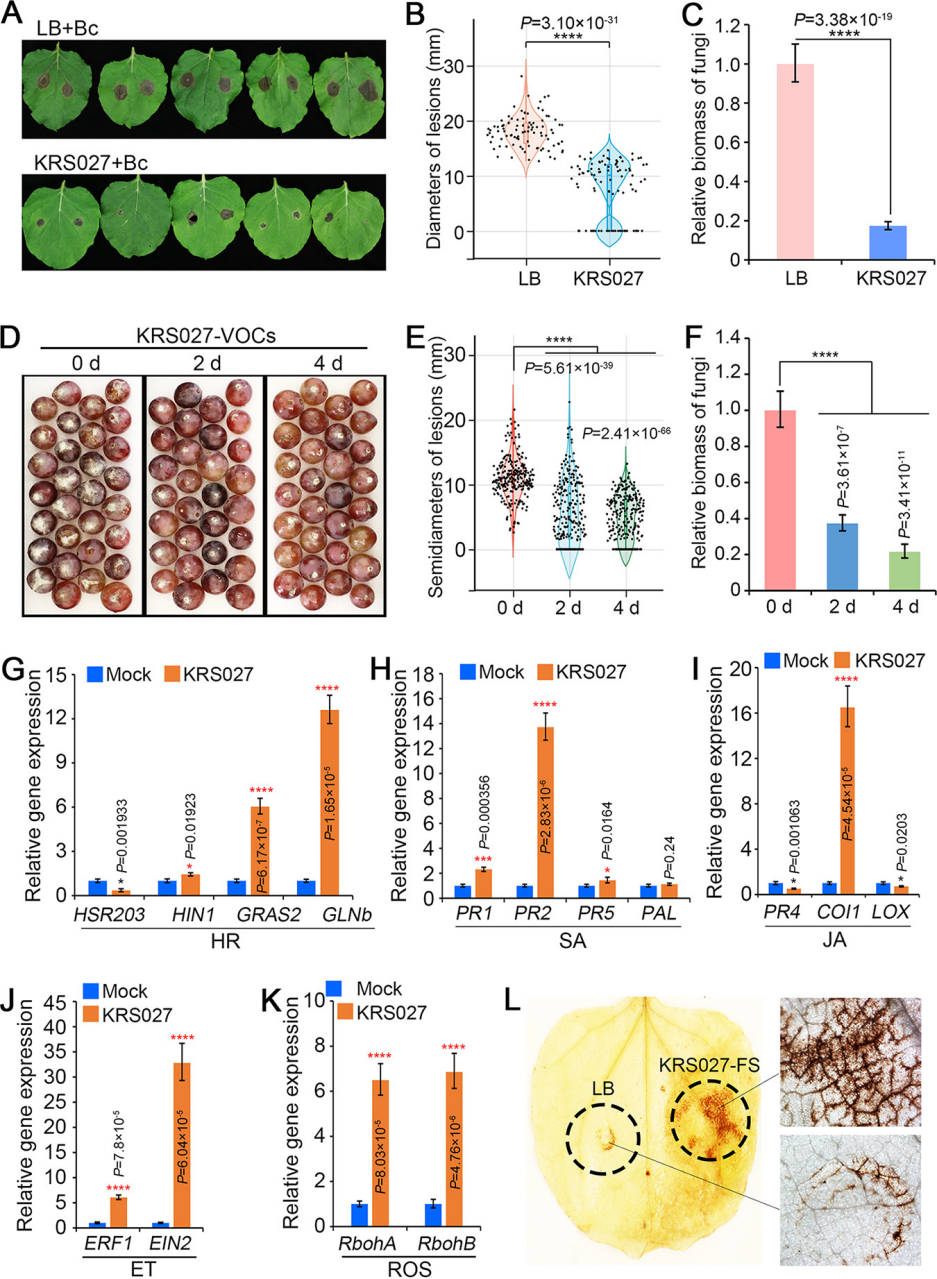
The strain KRS027 reduced the pathogenicity of B. cinerea on N. benthamiana leaves and table grapes. (A) Disease symptoms of B. cinerea on N. benthamiana leaves that were first treated by KRS027 fermentation broth. Spraying of Luria-Bertani (LB) broth was used as control. The phenotypes were collected at 3 days after B. cinerea infection. (B) The lesion diameter of B. cinerea on N. benthamiana leaves were evaluated at 3 days after inoculation. (C) Fungal biomass of B. cinerea on N. benthamiana leaves was determined by quantitative PCR (qPCR). (D) Disease symptoms of B. cinerea on table grapes that were treated by volatile organic compounds (VOCs) released from KRS027. The treatment of LB plate was used as control. (E) The lesion diameters of B. cinerea on table grapes were evaluated at 4 days after B. cinerea infection. (F) Fungal biomass on table grapes of B. cinerea was determined by qPCR. (G to K) Relative expression of marker genes in N. benthamiana leaves treated with KRS027 detected by qPCR. (G) Defense-related genes involved hypersensitivity response (HR). (H) Salicylic acid (SA). (I) Jasmonic acid (JA). (J) Ethylene (ET) signaling pathways. (K) Marker genes (*NbRbohA* and *NbRbohB*) of reactive oxygen species (ROS). (L) ROS accumulation after injection of KRS027 fermentation supernatant (KRS027-FS) in N. benthamiana leaves from 4-week-old plants were determined by 3,3′-diaminobenzidine (DAB) staining. Error bars represent standard errors. *, **, ***, and **** represent significant differences at *P* < 0.05, *P *< 0.01, *P *< 0.001, and *P* < 0.0001, respectively, between the treatment and control group according to unpaired Student’s *t* test.

The role of isolate KRS027 in suppressing plant-pathogenic fungi, especially B. cinerea *in vivo*, was further investigated. Isolate KRS027 fermentation broth was sprayed on 5-week-old N. benthamiana leaves 12 h before B. cinerea inoculation and further incubated for 3 days. The results showed that KRS027 reduced the severity of gray mold caused by B. cinerea, with some leaves even having no disease symptoms compared to spraying with LB broth control (Fig. 4A). Accordingly, the lesion diameters of gray mold disease on tobacco leaves treated by KRS027 fermentation were significantly lower than the control (Fig. 4B). Fungal biomass was analyzed by quantitative PCR (qPCR). The results revealed that isolate KRS027 fermentation broth resulted in reducing fungal growth significantly compared to the LB broth-spraying control (Fig. 4C).

This study confirmed that isolate KRS027 could produce VOCs to inhibit fungal growth, especially against B. cinerea *in vitro* (Fig. 2D). We further investigated the control effect of VOCs released from KRS027 against grape gray mold development on Vitis labrusca × *vinifera* “Kyoho.” The VOCs produced by KRS027 that were grown for 2 or 4 days exhibited a major biocontrol effect, which was embodied in reduced disease symptoms, and significantly smaller lesions and fungal biomass compared with the control (Fig. 4D to F). VOC fumigation time had a certain positive effect on the grape gray mold control. The effect of fumigation after 4 days was better than that after 2 days of colonization, including the reduction of gray mold lesions and fungal biomass (Fig. 4E and F).

Previous studies have shown that biocontrol microorganisms could trigger plant immunity and induce systemic resistance (ISR) by secretion of secondary metabolite and VOCs, which improve the ability of plants to resist biotic stress as pathogenic fungi. To study whether KRS027 could trigger the plant defense response, the related marker genes expression levels of hypersensitivity response (HR), salicylic acid (SA), jasmonic acid (JA), and ethylene (ET) signaling pathway were examined by reverse transcription quantitative PCR (RT-qPCR). Compared with the control of LB broth-treated tobacco leaves, the marker genes of HR (*NbHIN1*, *NbGRAS2*, and *NbGlnb*) (Fig. 4G), SA (*NbPR1*, *NbPR2*, and *NbPR5*) (Fig. 4H), JA (*NbCOl1*) (Fig. 4I), and ET (*NbERF1* and *NbEIN2*) (Fig. 4J) signaling pathways were induced significantly in the tobacco leaves 2 days after spraying KRS027 fermentation broth. A plant’s systemic resistance is often accompanied by reactive oxygen species (ROS) accumulation. As expected, ROS-related marker genes *NbRobhA* and *NbRobhB* were highly upregulated 2 days after spraying with KRS027 fermentation broth (Fig. 4K). In addition, KRS027 fermentation supernatant injected into tobacco leaves was found to trigger ROS accumulation, which was detected by 3,3′-diaminobenzidine (DAB) staining (Fig. 4L). Given the above results, KRS027 could effectively inhibit the occurrence of gray mold caused by B. cinerea by triggering plant innate immunity, including ISR, which is probably induced by SA, JA, and ET signaling pathways.

### KRS027 can directly affect B. cinerea.

**FIG 5:**
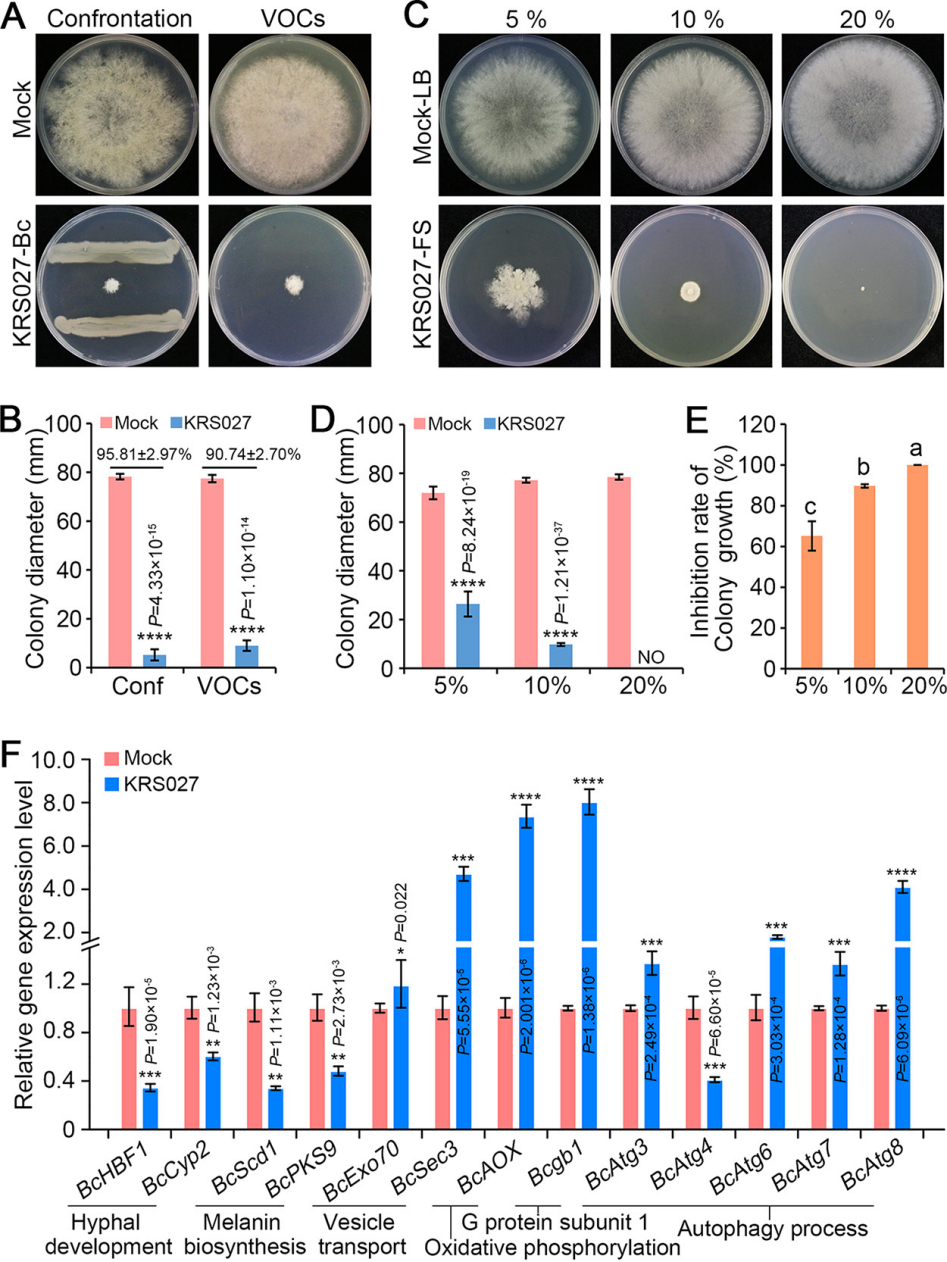
Isolate KRS027 effect on the growth and development of B. cinerea. (A) Antifungal activity of confrontation culture and covering fumigation of KRS027 against B. cinerea grown on potato dextrose agar (PDA) plates. (B) Diameters of colony growth at 5 days postinoculation (dpi). The numbers above the columns indicate the inhibition rates. (C) The inhibition activity of different concentrations of KRS027 fermentation supernatant (KRS027-FS) against B. cinerea on PDA plates. (D) Diameters of colony growth at 5 dpi on PDA plates containing the KRS027 fermentation supernatant (KRS027-FS) of different volume ratio. (E) The inhibition rate of KRS027-FS against B. cinerea were evaluated at 5 dpi. (F) Relative expression of marker genes of B. cinerea grown on a dual confrontation culture at 3 dpi. The genes were related to hyphal development (*BcHBF1* and *BcCyp2*), melanin biosynthesis (*Bcscd1* and *BcPKS9*), vesicle transport (*BcSec3* and *BcExo70*), G protein subunit 1 (*Bcgb1*), mitochondrial oxidative phosphorylation (*BcAOX*), and autophagy (*BcAtg1*, *BcAtg3*, *BcAtg4*, *BcAtg6*, *BcAtg7*, and *BcAtg8*). Error bars represent standard errors. **, ***, and **** represent significant differences at *P *< 0.01, *P *< 0.001, and *P* < 0.0001, respectively, according to unpaired Student’s *t* test.

The dual confrontation culture assay and two sealed base plates (VOCs) assay indicated that KRS027 could inhibit colony growth and expansion of B. cinerea on potato dextrose agar (PDA) plates. The inhibition rates of VOCs and secondary metabolites are 95.81% and 90.74%, respectively (Fig. 2A and B). The supernatant broth was prepared after KRS027 was shaken in LB liquid medium at 28°C for 3 days. The antifungal effect of the KRS027 supernatant in PDA plates was apparent in a concentration-dependent manner at 5%, 10%, and 20%. The B. cinerea colony had hardly grown on the PDA plates containing 20% KRS027 supernatant (Fig. 5C). The KRS027 supernatant inhibited the fungal colony growth and affected the colony phenotypes on plates (Fig. 5D and E). Moreover, the inhibition rate of extracellular metabolites against B. cinerea was dose-dependent, which was positively correlated with the concentration of supernatant (Fig. 5E).

The relative transcript levels of genes involved in B. cinerea growth and development were detected under dual confrontation culture between KRS027 and B. cinerea by RT-qPCR. The genes *BcHBF1* and *BcCyp2* engaged in the process of hyphal development, and both *BcScd1* and *BcPKS9* involved in melanin biosynthesis were downregulated significantly after 3 days at dual confrontation culture (Fig. 5F). On the contrary, the vesicle transport-related genes *BcSec3* and *BcExo70*, the G protein β subunit gene *Bcgb1*, and the mitochondrial oxidative phosphorylation-related gene *BcAOX* were significantly upregulated in response to KRS027 VOCs (Fig. 5F). Autophagy is a process of controlling cellular degradation including senescent protein and organelles degradation to maintain homeostasis in eukaryotic cells. The relative expression levels of autophagy-associated genes of B. cinerea were also influenced in response to biotic stress caused by KRS027, of which *BcAtg3*, *BcAtg6*, *BcAtg7*, and *BcAtg8* were upregulated and *BcAtg4* was downregulated (Fig. 5F). Taken together, the strain KRS027 had a direct antifungal activity that was caused by both supernatant compounds and VOCs. The antifungal mode of action by the secreted compounds interfered with the processes of hyphal development and melanin biosynthesis, while it affected the vesicular transport and the autophagy process.

### KRS027 disturbs the cellular structure of B. cinerea.

**FIG 6:**
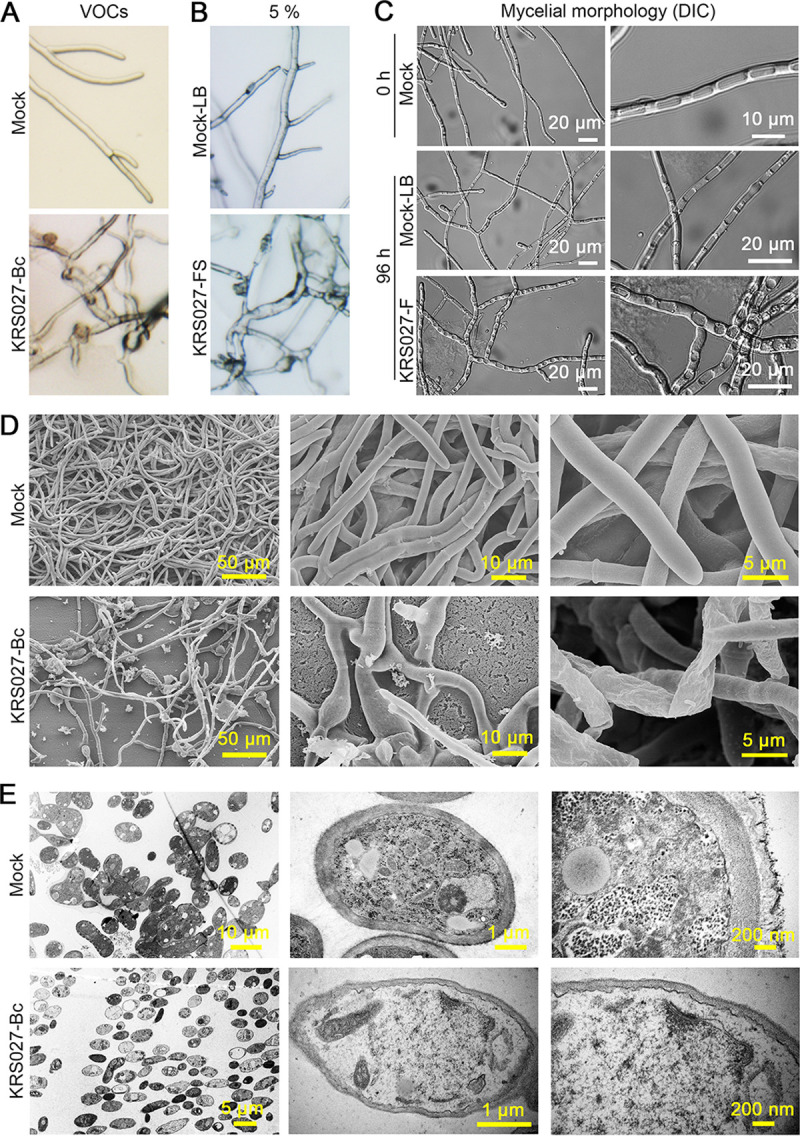
Microscopic morphology observation for B. cinerea treated by KRS027. (A, B) The mycelium morphology of B. cinerea grown in the presence of volatile organic compounds (VOCs) released from KRS027 or 5% KRS027 fermentation supernatant (KRS027-FS), respectively. Clasping with Luria-Bertani (LB) plate or 5% LB broth was used as the negative control, respectively. (C) The mycelium morphology of B. cinerea in the presence of KRS027 fermentation broth for 96 h was observed by a differential interference contrast microscope (DIC). The mycelium incubated in LB broth was set as a control. (D, E) Scanning electron microscope (SEM) and transmission electron microscope (TEM) were used to examine the further hyphal morphology defects from the edges and superficial state and the clear outline and cell internal state of the inhibitory zone of B. cinerea due to the secondary metabolites and VOCs of isolate KRS027, respectively. The bars represent the actual sizes.

The hyphal developmental morphology of B. cinerea was observed by stereomicroscope on PDA plates with 5% supernatant (extracellular metabolites of KRS027) or VOCs produced from KRS027. The results presented that the hyphal morphology of the treated group showed enlargement, deformation, disorderly growth, and aberrant dendritic branch, while hyphae from the control group were intact and had normal radial growth (Fig. 6A and B). In cases of coincubation between KRS027 fermentation and B. cinerea hyphae for 4 days at 25°C, hyphal morphology was observed using a differential interference contrast microscope (DIC). We observed that the outline of B. cinerea hyphae treated by KRS027 was swollen, rough, and irregular compared with the control (Fig. 6C). Scanning electron microscope (SEM) and transmission electron microscope (TEM) were used to examine any morphological defects of B. cinerea from the edges of the inhibitory zone of the dual confrontation culture after 3 days. An apparent damage, distortion, and intracellular organelles collapse of fungal cells was apparent in the presence of KRS027 (Fig. 6D and E). The surface of normal hyphae was smooth, and the outline was unambiguous, while the hyphae treated by KRS027 were grievously swollen and enlarged, accompanied by some of the hyphae cell walls being rough and shriveled (Fig. 6D). The SEM analysis revealed that the normal growth hyphae had distinct cell wall, plasma membrane, nucleus (karyotheca and nucleolus), and organelles (mitochondria, endoplasmic reticulum, and Golgi apparatus); and the cytoplasm contained a large amount of glycogen and apparent lipid droplets (Fig. 6E). In comparison, hyphae treated with KRS027 had extreme leakage of cytoplasm as glycogen disappeared and organelles collapsed. In addition, the microscopic observations also demonstrated that there was a plasmolysis phenomenon and even plasma membrane damage, and the cell wall became thicker than control (Fig. 6E). Together, these results indicated that the secreted metabolites of KRS027 had a direct antifungal function and could eventually lead to hyphae deformation, degradation of the fungal cell wall, plasma membrane damage, collapse of organelles, and leakage of cell contents, which suggested that the isolate KRS027 and its extracellular metabolites and VOCs had quite a strong direct inhibition activity against pathogenic fungi as B. cinerea.

### Crude extract of KRS027 fermentation against B. cinerea.

**FIG 7:**
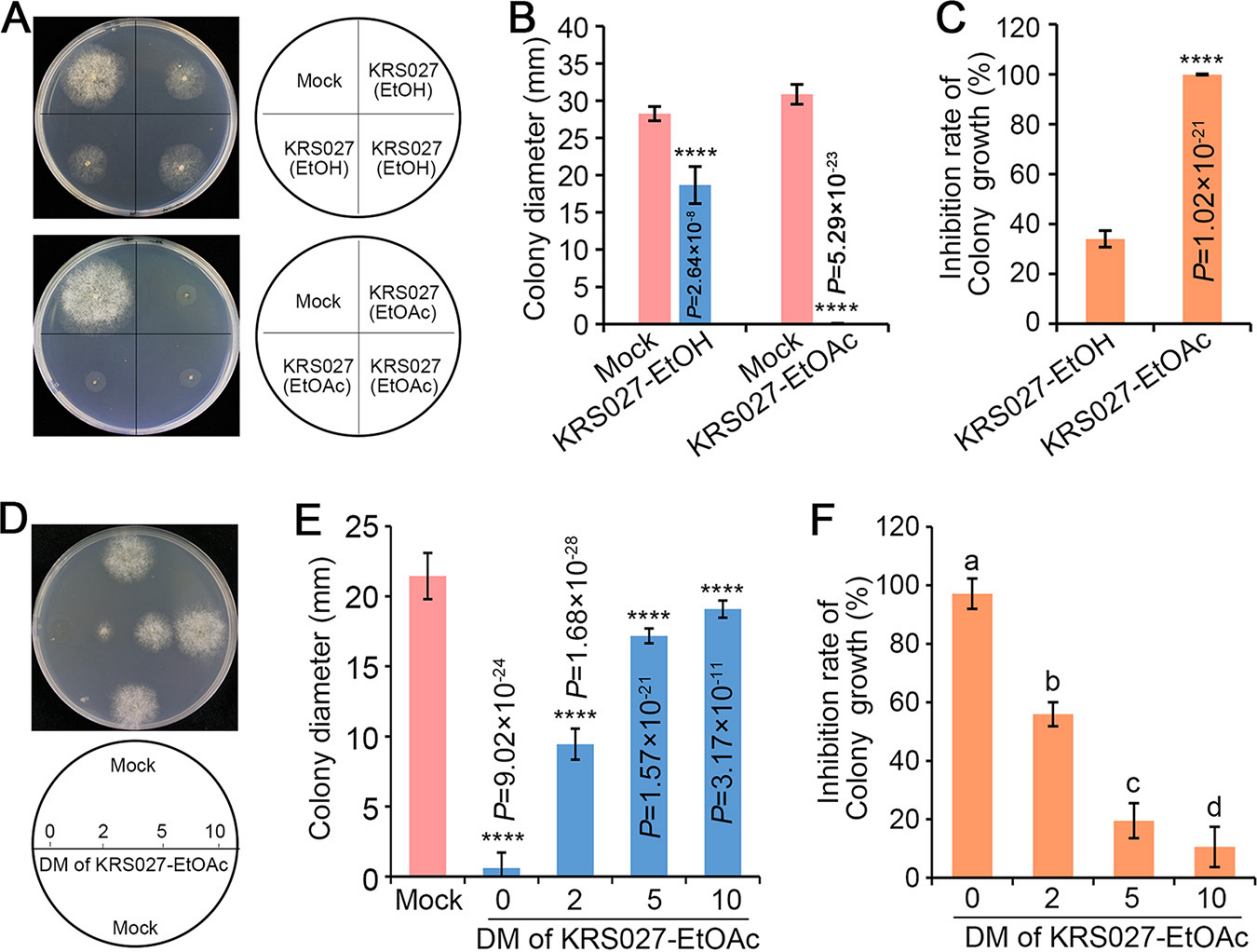
Crude extract of KRS027 fermentation supernatant inhibits B. cinerea. (A) The active substances of KRS027 fermentation supernatant (KRS027-FS) were extracted by ethyl alcohol (EtOH) and ethyl acetate (EtOAc), and the suppression activities against B. cinerea were detected. (B, C) The colony diameters and inhibition rate of KRS027-FS crude extract against B. cinerea were evaluated at 60 h postinoculation (hpi). (D) The antifungal capacity of EtOAc KRS027-FS crude extract against B. cinerea at 2, 5, and 10 dilutions, respectively. (E, F) The diameters of the colony and inhibition rate of ethyl acetate (EtOAc) crude extract at different dilutions against B. cinerea were evaluated at 48 hpi. Error bars represent standard errors. ****, significant differences at *P* < 0.0001 according to unpaired Student’s *t* test. The letters above the columns represent significant differences at *P *< 0.01 according to one-way analysis of variance (ANOVA) and LSD.

Ethyl alcohol (EtOH) and ethyl acetate (EtOAc) were used to extract crude proteins/peptides and secondary metabolites from the fermentation supernatant broth of KRS027. To test the antifungal activity, KRS027 crude extract was redissolved in methyl alcohol (MeOH) and was dripped on the PDA plates; the 1 × 1-mm^2^ hypha patch of B. cinerea was inoculated in the center of the region where the crude extract was previously added. The results demonstrated that both EtOH and EtOAc crude extracts had antifungal activity to various degrees, which was reflected in the growth phenotypes and radial growth diameters of the B. cinerea colony (Fig. 7A and B). It is worth mentioning that the inhibition rate of the crude extract with EtOAc was 99.86% and was much more effective than EtOH extract (33.98%) (Fig. 7C). The suppression effect of EtOAc crude extract against B. cinerea was dose dependent (Fig. 7D to F). These results indicated that the antifungal activity is also strongly affected by the extracellular secondary metabolites.

## DISCUSSION

The genus Burkholderia sp. is widespread in the natural environment; it is not only a soil inhabitant but also exists in the rhizosphere and endophytically colonizes plants, and some strains were also found in animals and humans (23). Based on the phylogenetic analysis of 16S rRNA gene sequences, 55 Burkholderia species are divided into several major groups, including the pathogenic group, the plant-beneficial group, and the environmental group (23). The pathogenic group can cause animal, human, and plant diseases, including Burkholderia cepacia complex (Bcc), which is involved in closely related bacterial species and Burkholderia pseudomallei members (23, 36). Nevertheless, nonpathogenic members of Burkholderia species have great potential for biological control agents and biofertilizers for agricultural applications (4, 37). In this study, we isolated an antagonistic strain KRS027 from the rhizosphere soil of healthy plants in a pathogen-infested cotton field. The isolated strain was identified as B. gladioli by MLSA-MLST analysis of tandem loci of *atpD*, *gltB*, *gyrB*, and *trpB* (Fig. 1D). This identification strategy was performed because the sequence variation within 16S rRNA is insufficient to differentiate specific and closely related species (34, 35).

The B. cepacia complex (Bcc), comprising at least 17 different but closely related species, was reported to emerge in the 1980s as a human pathogen (38). Although B. gladioli belongs to one member of Bcc and was reported to have pathogenicity activity for plants and humans (23), recent studies indicated that some B. gladioli isolates have not caused any disease symptoms to plants, of which some isolates even were proven to have the capacity to inhibit various plant-pathogenic fungi, particularly in the aspect of fungal disease, and to confer the plant growth promotion effect (37). In this study, B. gladioli KRS027 was identified to have a broad spectrum antifungal effect against B. cinerea, V. dahliae, M. oryzae, Fusarium sp., and Colletotrichum sp. (Fig. 2 and 5). Recent advances also displayed that B. gladioli have an antagonistic function against plants and human pathogens, particularly in fungal disease. KRS027 also was found to be innocuous and have no phytotoxic effect on tobacco leaves. Furthermore, KRS027 does not break down the red blood cells by hemolysis test (Fig. 3A and B). Thus, B. gladioli KRS027 seems to be safe for plants and humans. The innocuousness and safety of isolated strains are essential preconditions for proceeding with potential biocontrol microbial resources or biofertilizers (39). Thus, it is necessary to further determine the pathogenicity of KRS027 for humans, such as the acute toxicity test using limited methods and heredity toxicity examinations, including the Ames test, marrow micronucleus test, and sperm shape abnormality test, etc.

Some nonpathogenic Burkholderia species have characteristics related to PGP, including nitrogen fixation, phosphate, and potassium solubilization, siderophores generation, indole acetic acid (IAA), and 1-aminocyclopropane-1-carboxylate (ACC), deaminase production, and rhizosphere competence by secretion of allelochemicals (13, 23, 31, 40). A whole-genome sequencing draft of Burkholderia remsis BE51 that possesses antifungal activities against various fungi contained 14 putative secondary metabolites biosynthetic clusters, such as bacteriocin, siderophores, and nonribosomal peptides. Additionally, genes involving IAA production and ACC deaminase were also predicted, which reflected that the B. remsis BE51 strain was a promising microbial resource for biocontrol and PGP (41). In addition to their antagonistic activity, B. gladioli strains are also generally associated with PGP function (23, 31). Likewise, KRS027 could solubilize phosphates and potassium, produce siderophore, and have a nitrogen fixation function (Fig. 3C and D), which offered competitive advantages for biological control application and suggested that B. gladioli KRS027 might have a PGP function.

Degrading the phytopathogen’s cell wall is important for biocontrol bacteria to exert antifungal and mycophagy function (42). Burkholderia species were reported to secrete some hydrolytic and catalytic enzymes associated with the degradation of the fungal cell wall and feed on fungal hyphae (31). The target strain KRS027 was detected to have various catalytic enzymes activities, including gelatinase, proteinase, amylase, catalase, urease, and phenylalanine deaminase (Fig. 3E; Table S2) that may be involved in pathogen cell wall degradation. Indeed, the results of SEM and TEM observation demonstrated that the hyphae cell wall of B. cinerea in dual confrontation culture with KRS027 were deformed, degraded, and even destroyed (Fig. 6), which is probably related to the various cell wall degradation enzymes and other catalytic activities of KRS027.

It is well known that autophagy is a conserved cellular degradation process that maintains cell homeostasis at basal conditions, acts as a survival mechanism during stress conditions, and plays an important role in the normal differentiation and development of cells (43, 44). In the study of yeast, autophagosome formation was found to be a key step for the autophagy process, which includes two ubiquitin-like (UBL) conjugation systems as the Atg8-Atg12 complex (45). The B. cinerea mutants *BcAtg3* and *BcAtg7* do not have the autophagy process (46); *BcAtg6* was related to infection hyphae structure morphogenesis (47), and cysteine protease *BcAtg4* determined its crucial roles in the deconjugation step of conjugation of phosphatidyl ethanolamine (PE) to UBL Atg8 proteins (48). In this study, autophagy-related genes expression of B. cinerea were up-regulated (*BcAtg3*, *BcAtg6*, *BcAtg7*, and *BcAtg8*) or down-regulated (*BcAtg4*) under the conditions of dual confrontation culture between KRS027 and fungi (Fig. 5F), which suggested that KRS027 may affect hypha growth and development of the fungi by disturbing its autophagy process to maintain the balance of cellular homeostasis under biotic stress.

Fungal melanin plays an important role in fungal survival and competition to withstand extreme temperatures, ultraviolet (UV) radiation, and osmotic and oxidative stress (49–51). In B. cinerea, melanin accumulation is beneficial to the survival of conidia and microsclerotia, of which *Bcscd1* is required for its biological process (52). The polyketide pathway mediated by PKS gene clusters is highly conserved in the melanin synthesis pathway (53). Marker genes (*Bcscd1* and *BcPKS9*) involved in melanin synthesis were significantly suppressed under the condition of dual confrontation culture between KRS027 and fungi (Fig. 5F). Thus, the inhibition of melanin formation by KRS027 could also be one of the possible mechanisms for direct fungal control (44, 54).

Until now, an increasing number of antagonistic bacteria such as Bacillus, Pseudomonas, Burkholderia, Klebsiella, and Paenibacillus species have been proven to effectively control crops and postharvest disease effectively. In this research, B. gladioli KRS027 inhibited the pathogenic fungi B. cinerea growth on PDA plates (Fig. 2 and 5), tobacco leaves, and fruits of table grapes (Fig. 4A to F) by direct antagonism or by secreting secondary metabolism and VOCs both *in vitro* and *in vivo*. Furthermore, biocontrol activity by antagonistic bacteria against fungal pathogens could be by direct inhibition or by triggering plant immunity. In response to pathogens, the plant can induce systemic acquired resistance (SAR) to increase the resistance of plants against various pathogens (55, 56). Secondary metabolites and VOCs generated from antagonistic bacteria could also activate the SA-dependent SAR of plants (13, 14, 57). For instance, Bacillus cereus AR156 can activate the SAR response by triggering the SA signal pathway (58).

Our research found that spraying the tobacco leaves with KRS027 fermentation broth increased the expression of SAR marker genes such as *NbPR1*, *NbPR2*, *NbPR5*, and *NbPAL*, which suggested that KRS027 stimulates the plant’s SAR (Fig. 4H). At the same time, the marker genes of reactive oxygen species (ROS) accumulation, NADPH oxidase (NbRbohA and NbRbohB), were also significantly upregulated (Fig. 4K), which could lead to the activation of the SA-dependent SAR (13, 58). Another plant defense response is mediated by JA and ET (14). Surprisingly, RT-qPCR analysis results showed that not only was the SA-dependent defense response upregulated (Fig. 4G and H), but also the transcriptional levels of JA/ET-related marker genes were increased under the treatment of KRS027 fermentation broth (Fig. 4I and J). These results indicate that KRS027 may induce both SAR and ISR, which are regulated by SA and JA/ET, respectively. Recent research has shown that SA- and JA/ET-dependent signaling pathways are involved in ISR induced by biocontrol bacteria in plants. For instance, Bacillus amyloliquefaciens CRN9 inhibits pathogen colonization by inducing ISR that was related to both SA- and JA/ET-dependent signaling pathways (59); B. velezensis CLA178 enhanced gene expression of both SA and ET signal pathways, which mediated ISR in plants (60). Combined with our results, this research seemed to show that SA- and JA/ET-dependent signaling pathways may simultaneously participate in biocontrol bacteria-mediated ISR in plants. Therefore, SA- and JA/ET-dependent signaling pathways may be involved in B. gladioli KRS027-induced ISR.

Burkholderia species have shown a pathogen inhibition function by secondary metabolites such as pyrrolnitrin, phenazines, siderophores, nonribosomal peptides and polyketides, and phenazine-1-carboxylic acid (41). The isolate KRS027 has a strong fungal inhibition activity that is related to the extracellular secretion of proteins/peptides and secondary metabolites and VOCs. Secondary metabolites extracted by EtOAc showed an antifungal activity significantly better than the EtOH extract (Fig. 5 and 7), indicating the potential of secondary metabolites of organic origin of KRS027 in antifungal activity. The study of specific antagonistic compounds of specific secreted metabolites and VOCs should be further investigated in future work.

In conclusion, the strain KRS027, isolated from the rhizosphere soil of a healthy plant from a pathogen-infested cotton field, was identified as B. gladioli by morphological identification, phylogenetic analysis, and physiobiochemical characteristics. This strain has a broad spectrum of antifungal activity against various fungal pathogens and is innocuous to plants, animals, and humans. The B. gladioli KRS027 strain demonstrated PGP traits *in vitro*. Furthermore, KRS027 can effectively reduce the occurrence of gray mold in tobacco leaves and table grapes. The possible mode of action of this strain includes fungal cell wall degradation, inhibition of melanin biosynthesis, and disturbing the autophagy process. On the other hand, the KRS027 strain could trigger a wide plant immune response. In summary, KRS027 is likely to be a promising resource for biocontrol agents and biofertilizers. Characterization of its antagonistic mechanisms will provide a basis for the targeted development of biocontrol products based on this strain.

## MATERIALS AND METHODS

### Growth of microbes and plant material.

The B. gladioli strain KRS027, isolated from healthy plant rhizosphere soil in a pathogen-infested cotton field from Xinjiang, China, was cultured in Luria-Bertani (LB) broth (10 g/liter tryptone, 5 g/liter NaCl, 5 g/liter yeast extract, 1,000 mL double-distilled H_2_O [ddH_2_O]) at 28°C. B. cinerea strain B05.10 were cultured on potato dextrose agar (PDA) medium (200 g/liter potato, 20 g/liter glucose, 15 g/liter agar, 1,000 mL ddH_2_O) at 25°C. Tobacco seedlings (N. benthamiana LAB) were grown in a greenhouse with 16-h light/8-h dark photoperiods at 25°C for 4 weeks before assessing the control efficiency assays. Each age-appropriate tobacco plant was cultivated in advance in a pot (10-cm diameter and 8-cm height), with a nursery substrate mixture of nutrient soil and vermiculite at 1:l (vol/vol). The watering regime was carried out once every 2 days, and the watering amount was subject to the surface wetting degree after nursery substrate absorption. Throughout the growing period, these tobacco plants were cultivated in a fixed position in the greenhouse, while different batches of tobacco seedlings might be grown in different parts of the same greenhouse. Each experiment was performed with same batch of tobacco seedlings.

### Gram staining of bacteria.

The strain KRS027 was stained by a series of steps, including initial dyeing, mordant dyeing, decolorization, and redyeing as described previously (61). Pseudomonas alcaligenes KRS022 (Gram-negative) and B. velezensis KRS001 (Gram-positive) were used as controls (our unpublished data).

### Multilocus sequence analysis and typing.

The identification of isolate KRS027 was studied by multilocus sequence analysis and typing (MLSA-MLST) according to the previously described method with modifications (34). Briefly, the genomic DNA of KRS027 was extracted using a TIANamp bacteria DNA kit (Tiangen, Beijing, China) following the manufacturer’s instructions. Four housekeeping genes (*atpD*, *gltB*, *gyrB*, and *trpB*) of each studied strain were amplified using the specific primers listed in Table S1; they were sequenced and then aligned to the reference genome of Burkholderia sp. strains from the National Center for Biotechnology Information (http://www.ncbi.nlm.nih.gov/genomes/lproks.cgi) and the B. cepacia complex PubMLST (https://pubmlst.org/bcc/) database followed by constructing a phylogenetic tree using MEGA11 (62).

### *In vitro* antifungal activity assays.

The *in vitro* antifungal activity of KRS027 against seven pathogenic fungi was assessed using confrontation culture assay. In brief, a 6-mm diameter mycelial disc from a 5-day pathogenic fungus was inoculated on a PDA plate, and the cell suspension of KRS027 (optical density at 600 nm [OD_600_] = 1.0) was drawn as a straight line at one side of the mycelial disc with a distance of 20 mm. The LB broth was used as the control. The plates were incubated at 25°C for 5 days. Each treatment had at least five biological repeats, and the assay was replicated twice. The radial distance between the center of the disc and the colony’s outer edge was measured. The distances of KRS027 cell suspension-treated and LB broth-treated are denoted as “a” and “b,” respectively. The related inhibition rate (IR) was calculated using the formula: IR (%) = (1 - [*b* - 2]/[*a* - 2]) × 100%.

The antifungal activity of the VOCs released from KRS027 against seven fungal pathogens was evaluated by the two sealed base plate assays as described previously (18). The 50 μL cell suspension of KRS027 (OD_600_ = 1.0) was coated on LB plate, and a 6-mm diameter mycelial disc of pathogenic fungi was inoculated on a PDA plate. Then, the two treated plates were sealed together with Parafilm and incubated at 25°C for 5 days. The two sealed base plates, involving a LB plate with 50 μL LB broth and a PDA plate with pathogenic fungi, respectively, served as control. All treatments and controls were performed with five replicates, and the assay was performed twice. The diameter of the fungal colony for each plate (the colony diameters of treatment and control groups are denoted as “c” and “d,” respectively) was measured, and the inhibition rate (IR) was calculated using the following formula: IR (%) = (1 - [*d* - 4]/[*c* - 4]) × 100%.

### Innocuous and safety evaluation of KRS027.

To test whether strain KRS027 is harmless to plants, its cell suspension (OD_600_ = 4.0) was prepared and sprayed on 4-week-old tobacco leaves. The leaves were sprayed with sterile water, and LB broth served as the control. Three leaves from different plants were detected in each treatment. Plant leaves and mesophyll cells were characterized using a stereomicroscope Nikon SMZ18 after 2 days of treatment. The experiment was repeated three times with similar results.

The hemolysis of KRS027 was evaluated by culturing the strains on the commercial blood agar medium, including Mueller-Hinton (MH) plates and Columbia Blood plates at 28°C for 48 h as described previously. Escherichia coli DH5α with no hemolysis was used as a negative control (39). This assay was performed three times.

### *In vitro* PGP assays by KRS027.

The plant growth promotion traits of strain KRS027 were quantified by a series of experiments such as nitrogen fixation, phosphate and potassium solubilization, and siderophore production. A total of 10 μL of KRS027 strain suspension (OD_600_ = 1.0) was dropped on the different functional characteristic plates, which were incubated at 28°C for 4 days before observing the phenotypes. E. coli DH5α was used as a negative control. Each experiment included five biological repetitions and was repeated twice.

**(i) Nitrogen fixation ability assay.** Ashby’s medium, also known as nitrogen-free agar medium (0.2 g/liter KH_2_PO_4_, 0.2 g/liter NaCl, 0.2 g/liter MgSO_4_, 5.0 g/liter CaCO_3_, 0.1 g/liter K_2_SO_4_, 10.0 g/liter glucose, 15.0 g/liter agar, 1,000 mL ddH_2_O, pH 7.4) was used to determine the nitrogen fixation ability of KRS027.

**(ii) Phosphate solubilization ability assay.** Pikovskaya (PVK) medium (10.0 g/liter glucose, 0.5 g/liter [NH_4_]_2_SO_4_, 0.3 g/liter NaCl, 0.3 g/liter MgSO_4_, 0.03 g/liter MnSO_4_, 0.3 g/liter KCl, 0.03 g/liter FeSO_4_, 5.0 g/liter Ca_3_(PO_4_)_2_, 15.0 g/liter agar, 1,000 mL ddH_2_O, pH 7.4) with insoluble Ca_3_(PO_4_)_2_ was used to test the ability of KRS027 to solubilize phosphate.

**(iii) Potassium solubilization ability assay.** The potassium solubilization ability of KRS027 was determined using Alexandrov medium (5.0 g/liter sucrose, 0.5 g/liter MgSO_4_·7H_2_O, 0.1 g/liter CaCO_3_, 2.0 g/liter Na_2_HPO_4_, 0.005 g/liter FeCl_3_·6H_2_O, 1.0 g/liter glass powder/potash feldspar, 15 g/liter agar, 1,000 ddH_2_O mL) with insoluble glass powder or potash feldspar.

**(iv) Siderophore production assays.** Siderophore production of KRS027 was determined using blue agar medium containing Chrome Azurol S (CAS), hexadecyl-trimethyl-ammonium bromide (HDTMA), and iron ion. The basal medium and CAS solution were prepared in advance as previously described (63) with a few modifications. Basal medium includes 100 g glucose, 20 g peptone, 0.5 g MgSO_4_·7H_2_O, 0.5 g CaCl_2_, 20 g agar, and 1,000 mL ddH_2_O. A total of 100 mL of 10× CAS solution of each includes 0.06 g CAS, 0.0027 g FeCl_3_·6H_2_O, 0.073 g HDTMA, pH 6.8, and 1 M phosphatic buffer solution (PBS) buffer, pH 6.8. The basic medium, CAS solution, and PBS buffer were mixed in a ratio of 2:1:1.

**(v) Proteinase activity assay.** The specific medium (10 mL fresh skim milk, 0.5 g/liter yeast extract, 0.5 g/liter NaCl, 15 g/liter agar, 1,000 mL ddH_2_O, pH 7.2) was used to perform this assay.

**(vi) Amylase activity assay.** The strain KRS027 was inoculated in the inorganic salt medium with the soluble starch (1.0 g/liter K_2_HPO_4_, 1.0 g/liter MgSO_4_, 1.0 g/liter NaCl, 2.0 g/liter [NH_4_]_2_SO_4_, 2.0 g/liter CaCO_3_, 0.001 g/liter FeSO_4_, 0.001 g/liter MnCl_2_, 0.001 g/liter ZnSO_4_, 10.0 g/liter soluble starch, 1,000 mL ddH_2_O, pH 7.2) and shaken for 10 h at 28°C. Then, 10 μL Lugol’s iodine was added to the above fermentation broth of KRS027. The E. coli DH5α was used as a negative control.

### Biocontrol effect of KRS027 against plant disease.

Four-week-old tobacco seedlings were used to evaluate the biocontrol activity of isolate KRS027 against gray mold disease (B. cinerea). Either the KRS027 cell suspension (OD_600_ = 1.0) or LB broth (control) was sprayed on tobacco leaves and covered with an airtight clear dome at 25°C for 12 h before inoculation of 1 × 1-mm^2^ hypha patch of B. cinerea on both sides of tobacco leaves. The inoculated tobacco plants were placed in the same condition for 3 days before measuring the lesion diameters. This assay included at least 15 leaves from six tobacco seedlings in each treatment, and the experiment was repeated twice. The 20 × 20-mm^2^ tobacco leaves containing lesions of gray mold disease were collected, and the genomic DNA (gDNA) was extracted according to DNAsecure plant kit (Tiangen, Beijing, China) for the detection of relative fungal biomass. SYBR green-based qPCR was used to detect the biomass of B. cinerea with an initial 95°C denaturation step for 3 min, followed by denaturation for 15 s at 95°C, annealing for 20 s at 60°C, and extension for 20 s at 72°C for 40 cycles. The B. cinerea actin gene was used to quantify fungal colonization, and the N. benthamiana *EF-1α* gene served as an endogenous control. These primer pairs are listed in Table S1.

The VOCs released from KRS027 were used to prevent the occurrence of postharvest gray mold in grapes (Kyoho grapes, Vitis labruscana Kyoho). The strain KRS027 was spread on the LB plates and incubated at 28°C for 24 h. Then, these plates were placed without covers in an airtight container containing sterilized grapes, and one airtight container with four KRS027 plates, which were removed after 2 and 4 days, respectively. Table grapes with no visible wounds and no visible rotting were surface disinfected by immersion in 0.2% (wt/vol) hypochlorite sodium for 10 min, rinsed with sterile water at least five times, and air-dried on a sterile clean bench. The grapes were wounded using a syringe needle, and the 1 × 1-mm^2^ hypha patch of B. cinerea was inoculated on the wounds. Grapes that were not treated with VOCs were used as controls. The inoculated grapes were placed in the airtight container for 4 days before measurement of the lesion diameters. The grape skin was collected, and the total gDNA was extracted as described above for detecting B. cinerea biomass as described above. The B. cinerea actin gene was used to quantify fungal colonization, and the V. labruscana actin gene served as an endogenous control (Table S1).

### RT-qPCR.

For the detection of tobacco defense-related gene expression levels, the KRS027 fermentation broth (OD_600_ = 1.0) was sprayed on the 4-week-old tobacco leaves for 2 days before collecting samples. The LB broth treatment group was severed as a control. Total RNA extraction and first-strand cDNA were performed using the EASYspin Plus RNA speed extract kit (Aidlab, Beijing, China) and a cDNA synthesis supermix kit (TransGen, Beijing, China) according to the manufacturer’s instructions (both kits included a gDNA-removal procedure). Genes expression levels of hypersensitivity reaction (HR)-related genes (*NbHSR203*, *NbHIN1*, *NbGRAS2*, and *NbGLNb*), salicylic acid (SA) pathway-related genes (*NbPR1*, *NbPR2*, *NbPR5*, and *NbPAL*), ethylene (ET) pathway-related genes (*NbERF1*, *NbEIN2*), and ROS-related genes (*NbRbohA* and *NbRbohB*) were detected and normalized to the N. benthamiana *EF-1α* gene by RT-qPCR.

For detection of hypha growth and development-related gene expression levels, 5 μL KRS027 fermentation broth and a 1 × 1-mm^2^ hypha patch of B. cinerea were inoculated on an organic filter membrane on top of a PDA plate with a space of 15 mm. The fungal hypha was collected from the organic filter followed by total RNA extraction and synthesis of first-strand cDNA. The marker genes involved in hyphal development, melanin biosynthesis, vesicle transport, and autophagy processes were detected and normalized to the B. cinerea actin gene by RT-qPCR.

RT-qPCR was carried out using TransStart Top Green qPCR SuperMix (+DyeII) (TransGen, Beijing, China) following the manufacturer’s instructions and the procedures, including an initial 95°C denaturation step for 3 min, followed by denaturation for 15 s at 95°C, annealing for 20 s at 60°C, and extension for 20 s at 72°C for 40 cycles. The RT-qPCR experiment was repeated twice, and each contained three technical replicates. The relative transcript levels of different genes among various samples were evaluated using the 2^−ΔΔCT^ method as described previously (64). The RT-qPCR primer pairs are listed in Table S1.

### Microscopic observations.

After 4 days of treatment of VOCs and a 5% fermentation supernatant of KRS027, B. cinerea hypha was observed via a stereomicroscope (Nikon SMZ18). KRS027 fermentation broth, and B. cinerea hypha were coincubated for 2 days, and then the hypha was observed by a differential interference contrast microscope (DIC). The dual confrontation culture assays were performed for the observation of B. cinerea hypha. These hyphae were collected and fixed in a mixed solution with 2.5% glutaraldehyde and 4% paraformaldehyde. For scanning electron microscope (SEM) observations, the pretreated samples were dehydrated using graded ethanol at 30%, 50%, 70%, 80%, 90%, 95%, and 100%, respectively, and the following procedure was done by propylene oxide. The samples were dried using CO_2_ critical point dryer. For transmission electron microscope (TEM) observations, the samples were dried and embedded with epoxy and epoxypropane. The embedding blocks were cut into slices and adhesion to nickel mesh; then these slices were redyed by 2% lead citrate and 2% uranyl acetate.

### Antifungal activity of extract of KRS027 fermentation.

KRS027 was inoculated in LB liquid medium and shaken at 28°C for 4 days. The fermentation supernatant was collected by centrifuge at 4°C of 12,000 rpm and filtered with a 0.22-μm Millipore filter, and active compounds were extracted by organic solvent using absolute ethyl alcohol (EtOH) or ethyl acetate (EtOAc), individually. The EtOH was precooled at 4°C and added at a ratio of fermentation broth to EtOH 4:1 (vol/vol), and then both fermentation and EtOH were incubated at 4°C overnight. The crude proteins/peptides were collected after centrifuge at 4°C of 12,000 rpm and redissolved with Tris-HCl buffer (pH at 7.4). The fermentation broth ratio of EtOAc is 1:1 (vol/vol) and was incubated at room temperature after mixing until there was an obvious boundary and the organic and aqueous phases were very transparent. The organic phase was concentrated by rotary evaporation at 45°C and redissolved with absolute methyl alcohol (MeOH).

### Statistical analysis.

The standard errors in all involved figures were calculated for each treatment or group with at least three replicates. Unpaired Student’s *t* test was performed to determine statistical significance. One-way analysis of variance (ANOVA) and least significant difference (LSD) were used to analyze the statistical significance of multiple groups using SPSS version 23.

### Data availability.

The sequence data of phylogenetic analysis can be found in the National Center for Biotechnology Information database involved the reference genome of Burkholderia sp. strains (http://www.ncbi.nlm.nih.gov/genomes/lproks.cgi) and the B. cepacia complex PubMLST database (https://pubmlst.org/bcc/).
